# Melatonin Anticancer Effects: Review

**DOI:** 10.3390/ijms14022410

**Published:** 2013-01-24

**Authors:** Giuseppe Di Bella, Fabrizio Mascia, Luciano Gualano, Luigi Di Bella

**Affiliations:** 1 Di Bella Foundation, Via Guglielmo Marconi 51, Bologna 40122, Italy; E-Mail: brucebruce84@hotmail.com; 2 Private Laboratory of Physiology, Via Stefano Giovanni Marianini, Modena 41123, Italy; E-Mails: luciano@gualano.net (L.G.); luigidibella@libero.it (L.D.B.)

**Keywords:** melatonin, apoptosis, angiogenesis, APUD system, Di Bella Method

## Abstract

Melatonin (*N*-acetyl-5-methoxytryptamine, MLT), the main hormone produced by the pineal gland, not only regulates circadian rhythm, but also has antioxidant, anti-ageing and immunomodulatory properties. MLT plays an important role in blood composition, medullary dynamics, platelet genesis, vessel endothelia, and in platelet aggregation, leukocyte formula regulation and hemoglobin synthesis. Its significant atoxic, apoptotic, oncostatic, angiogenetic, differentiating and antiproliferative properties against all solid and liquid tumors have also been documented. Thanks, in fact, to its considerable functional versatility, MLT can exert both direct and indirect anticancer effects in factorial synergy with other differentiating, antiproliferative, immunomodulating and trophic molecules that form part of the anticancer treatment formulated by Luigi Di Bella (Di Bella Method, DBM: somatostatin, retinoids, ascorbic acid, vitamin D3, prolactin inhibitors, chondroitin-sulfate). The interaction between MLT and the DBM molecules counters the multiple processes that characterize the neoplastic phenotype (induction, promotion, progression and/or dissemination, tumoral mutation). All these particular characteristics suggest the use of MLT in oncological diseases.

## 1. Introduction: General Considerations on the Anticancer Effect of Melatonin

The functions of MLT involve numerous physiological processes, including circadian rhythm regulation, seasonal changes, sleep, reproduction and cardiovascular function [1]. MLT also modulates the functions of the immune and hemopoietic systems [2].

It is now accepted that MLT has marked dose-dependent antioxidative effect, providing protection against damage from carcinogenic substances, acting as a free radical scavenger [3]. This action can be reproduced experimentally, with important implications in the prevention and treatment of tumors. Numerous studies have tried to define the *in vitro* effects of MLT on the proliferation of tumor cell lines and on their apoptosis. There is no common agreement on why the action of MLT varies according to histological type, cell differentiation, sensitivity to oncogenic molecules and culture medium conditions [4–8].

The variability of MLT’s *in vitro* anticancer efficacy depends on the limitations and conditioning of the cell culture medium, without, obviously, the “biological context” and the complex and multifaceted interactions with which MLT exerts its anticancer properties *in vivo* [6]. In addition, the dynamics of division of normal cells and of tumor cells also depend on and are coordinated by a succession of MLT-correlated circadian time-markers [9].

Finally, MLT’s documented ability to negatively regulate both the transcription of the receptor gene of estrogen (ER) [10–12] and the oncogenic potential of the Growth Hormone (GH) axis with Prolactin-Insulin-like Growth Factor-1 (IGF-1) and of GH-dependent growth factors, such as Epidermal Growth Factor (EGF), Vascular Endothelial Growth Factor (VEGF), Fibroblast Growth Factor (FGF), Platelet Derived Growth Factor (PDGF), Transforming Growth Factor (TGF), or Hepatocyte Growth Factor (HGF), are aspects that certainly have an anticancer relevance [13–21].

## 2. The Main Direct Anticancer Mechanisms of Melatonin

### 2.1. Pro-Apoptotic

The direct anticancer action is exerted by inhibiting the proliferation and growth of tumor cells, thus hindering the tendency of healthy cells to become neoplastic, and inducing cellular turnover and replacement of tumor cells with healthy cells through apoptosis. The intrinsic, mitochondrial-dependent, activation route of caspases (cysteine-apartase) represents the “point of no return” towards the programmed cell death induced by MLT [22–24]. Numerous studies have documented the anticancer properties of MLT in solid tumors and in leukemia, with particular efficacy in lymphoproliferative tumors [25–28].

The use of MLT together with retinoic acid, on MCF-7 hormone-dependent breast cancer cells, showed a complete halt in cell growth and a reduction in the number of cells through apoptosis activation [29–32].

### 2.2. Antiproliferative

Various studies have shown that MLT has marked oncostatic properties that can reduce the promotion or progression of the tumor. Various authors have demonstrated that the antiproliferative properties of MLT take place through inhibition/blocking of the cell cycle [33–38].

This is confirmed by clinical studies in which, according to Luigi Di Bella, MLT alone cannot heal a tumor but without MLT it is difficult to heal any tumor. MLT therefore represents an absolutely necessary component in anticancer treatment, although it is not sufficient on its own [39–42].

Other studies have demonstrated the direct and selective inhibitory effect of melatonin on lymphoblastoid cell growth process [26–28]; El Missiry *et al*. studied the effect of MLT on Ehrlich ascites carcinoma cells (EAC), noting that it not only reduced their vitality and volume, increasing the survival of experimental animals, but also induced apoptosis of the EAC tumor cells [43].

A significant clinical fact emerged from a study on 250 patients with various forms of advanced and metastasized tumors, in whom the one-year survival rate and the objective tumor regression rate were much higher in the patients also treated with MLT compared to those who only received chemotherapy. Administration of MLT also significantly reduced thrombocytopenia, neurotoxicity, cardiotoxicity, stomatitis and asthenia [44].

Mediavilla, Sancez-Barcelo *et al.* observed an interesting oncostatic mechanism of action of MLT, through the activation and increase of p21/WAF1 and p53 suppressor genes which act by halting the reproduction cycle of tumor cells [45]. Human breast cancer cells (MCF-7) were studied *in vitro*, and it was found that, at physiological concentrations, MLT reduced the number and vitality of the tumor cells after 48 h. A year previously, a study was published on the effect of MLT, together with somatostatin, on murine colon cancer (colon-38), showing not only the antiproliferative effect but also an evident proapoptotic action [46].

### 2.3. Differentiating

At the Seventh Colloquium of the European Pineal Society at Sitges in 1966, several papers were presented on the oncostatic effect of MLT and its properties of inhibiting the metastatic spread of tumor cells. It was demonstrated that some oncogenes, including Rat sarcoma (RAS; Hras, Kras, NRas), are significantly inhibited by MLT [47]. Biochemical and molecular mechanisms of the oncostatic action of MLT also include the architecture of the cytoskeleton and the redox intracellular function. An important mediation mechanism of melatonin on the inhibitory action of the circadian-dependent growth of the tumor is the suppression of the epidermal growth factor receptor (EGFR) and of the activity of the mitogen-activated protein kinase, (MAPK) [9,48,49]. This takes place through the oxidation of linoleic acid and its conversion to 13-hydroxyoctadecadienoic (13-Hode) acid that can activate both EGFR and MAPK [50,51].

### 2.4. Anti-Angiogenetic

Other potential mechanisms concern the ability of melatonin to reduce tumoral angiogenesis, inhibiting the expression of the HIF-1alpha protein, inducing hypoxy in the cancer cells and acting on the Vascular Endothelial Growth Factor (VEGF) [52–56].

## 3. The Main Indirect Anticancer Mechanisms of Melatonin

### 3.1. Free Radical Scavenger Action

This counters carcinogenesis by means of free anti-radical and antioxidative effects [57–60]. This limits the toxicity of chemotherapy, simultaneously reinforcing the clinical response [61,62]. Chemotherapy causes an evident decrease in the serum levels of melatonin [63].

### 3.2. Myeloprotective/Myelostimulant Action

Myelosuppression represents a considerable problem in chemotherapy protocols. MLT protects the bone marrow and relative lymphoid tissues against the toxic effects of chemotherapy, and has a myeloprotective action with determining effects on blood composition, medullary dynamics and erythro-leuko-thrombocytopoiesis [61,62].

An essential fact discovered 30 years ago by Di Bella is the close functional interaction between MLT and platelets. This association is indispensable in understanding a number of phenomena essential not only for the physiology of blood, but of all tissues, in particular of the nervous system, both central and peripheral. The functional support of MLT is the platelet which carries it in structures of its cytoplasm, the “dense bodies”, where by means of a homeostatic mechanism it is mobilized on the basis of the plasma concentration [64–68].

**Figure 1:**
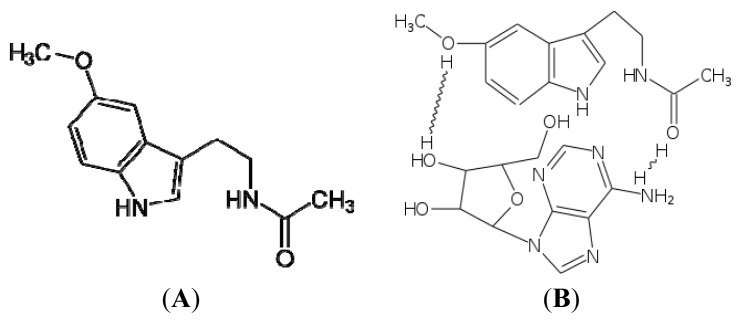
Insoluble in water (**A**), melatonin (**MLT**) dissolves in ethyl alcohol. Since absorption and bio-availability are linked with solubility, in the Luigi Di Bella formulation it is combined with a hydrogen bond to adenosine (**B**), thus becoming perfectly soluble and absorbable, with its biological-functional activities being reinforced. (Copyright Di Bella Foundation).

Conjugation with adenosine, though the hydrogen bond, according to the Luigi Di Bella formulation (Figure 1), makes MLT perfectly hydrosoluble and absorbable by the cell membranes. The platelets adhere to the wall of megakaryocytes and can release the melatonin already bound to adenosine. Melatonin can bind to ATP, ADP, AMP, polynucleic and ribonucleic acid and it is at this level that it exerts its antiblastic action [69–71].

### 3.3. Melatonin’s Action in Regulating the Immune System

MLT is involved in the body’s cell and humoral regulation, acting as an endocrine, autocrine and/or paracrine molecule [72]. This activity is sustained by its nuclear and membrane receptorial expression, with an intrinsic characteristic of the human lymphocyte populations. The existence of specific receptors for MLT in lymphoid cells confirms this indirect effect in regulating and reinforcing the immune response [73–75].

These protein binding sites have been described not only in human lymphocytes but also in granulocytes and in biological lymphoid reservoirs (thymus, spleen, bursa of Fabricius, *etc.*). The fundamental physiological role of MLT in the human immune system has thus been documented. Humoral regulation takes place through the production of cytokines in immunocompetent cells. MLT not only stimulates the production of natural killer cells, monocytes and leukocytes, but also increases the production of Interleukin 2-6-10-12 (IL-2-6-10-12) and Interferon-gamma (IFN-γ) by the mononucleate cells, promoting a T helper 1 (Th-1) lymphocyte response [25,76–79].

## 4. Mechanisms of Action and Physiology of Melatonin in Tumors

### 4.1. The Receptorial System

Although the molecule is highly diffusible and exerts systemic effects by means of at least two intracellular processes like modulation of the mitotic and cytoskeletal functions through the bond with calmodulin [80,81] and the free radical scavenger [82], two specific receptors have been identified: MT1 and MT2 [83,84]. Initially characterized at the level of the central nervous system, the receptors for MLT have been localized in all districts and cell types, including cells of the hemopoietic system such as lymphocytes, megakaryocytes, platelets, intestinal and prostatic cells, renal tubules, and cardiac miocytes [85–87]. Due to its chemical characteristics and low molecular weight (232, 278 kDa), MLT spreads easily both in extracellular liquid and in the cells themselves, in which orphan nuclear receptors have been identified [88]. From a chemical point of view, some of these nuclear receptors present structural similarities to retinoid receptors (ROR and RZR) [89,90] and the vitamin D receptor (VDR) [91,92].

These melatonin nuclear receptors are particularly widespread in the central nervous system, the main concentrations being in the pineal body, thalamus, hypothalamus, suprachiasmatic nucleus, cerebral cortex, superior colliculus of the lamina quadrigemina, habenulas, pars tuberalis, adenohypophysis and cerebellum [93–97]; a more or less ubiquitary presence of the melatonin receptors can be hypothesized, further confirming the primary role of MLT in vital functions. The chemical-metabolic properties linked to these receptors can help to understand some of the anticancer mechanisms of action of MLT.

Having also foreseen these recent findings, Di Bella suggested that the main anticancer effect of MLT consisted of the ubiquitary availability of the phosphor esters of AMP, ADP, and ATP [69,98,99].

**Figure 2:**
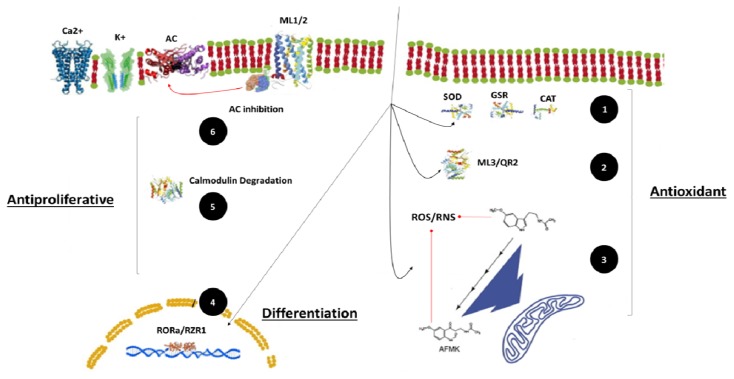
Anticancer action of melatonin: main molecular mechanisms. (1) Direct anti-oxidant enzyme activation; (2): bind with ML3 receptor; (3) direct antioxidant activity (*scavenger*); (4) gene expression regulation (differentiation); (5) calmodulin degradation: antiproliferative; (6) AC inhibition: antiproliferative. ML1/2: melatonin type receptor 1-2; SOD: super oxide dismutase; GRS: glutatione reductase; CAT: catalase; ML3/QR3: melatonin type receptor 3/quinone reductase 2; AC: adenylate ciclase; ROS: reactive oxygen species; RNS: reactive natrium species; AFMK: *N*(1)-acetyl-*N*(2)-formyl-5-methoxykynuramine. (Copyright Di Bella Foundation).

It is now accepted that MLT influences cell activity by acting mainly on the phosphor esters of adenosine and on other signal transduction systems, such as the protein C mediated inhibition of adenyl cyclase, inhibition of Ca^2+^ mobilization, inhibition of arachidonic acid release, action on protein kinase C, and opening of the potassium channels [100–107] (Figure 2).

### 4.2. Other Mechanisms

Melatonin can also exerts at different physiological levels its antitumoral properties by a set of complex mechanisms of action, not necessary involving the receptor pathway. These actions consist of apoptosis activation, inhibition of proliferation and cell differentiation (Figure 2). In fact, the intracellular redox state is strongly related to the MLT antiproliferative and cytotoxic actions in cancer cells. Therefore, tumor cell fate will depend on the ability of this indolamine to induce either an antioxidant environment—related to the antiproliferative effect or a pro-oxidant environment related to the cytotoxic effect (apoptosis). First, inhibition of proliferation is correlated with a decrease on intracellular reactive oxygen species (ROS) and an increase of the sub-cellular antioxidant enzymes (CAT, SOD and GRS levels), while induction of the programmed cell death is the result of the imbalance between ROS (increased) species production and antioxidant defenses (inhibited) [35]. The enzyme activation is also a crucial point for cell differentiation in several cancer cell lines [35,108]. Moreover, the same mechanisms can be reproduced by other well-known antioxidants molecules (retinoid, alpha-tocopheryl acetate, and ascorbic acid) [59].

### 4.3. The Amine Precursor Uptake and Decarboxylation System (APUD)

Kvetnoi *et al.* [109] confirmed the active role of MLT and of the molecules produced by the Amine Precursor Uptake and Decarboxylation system (APUD), both on tumor etiopathogenesis and proliferation and in antiblastic therapy. Analysis of the physiological characteristics of many biologically active substances produced by the Diffuse Neuro-Endocrine System (DNES) [110], such as melatonin, serotonin, gastrin, insulin, glucagon, somatostatin, *etc.*, confirms the important role of the hormones of these cells in the stages of tumor onset and proliferation, while the decrease in the number of these cells in the terminal stages of the tumor is significant [111].

Hormonal secretion in non-endocrine tumors has great theoretical and practical significance, confirmed by many authors, such as Maluf, Koerner and Bonkhoff [112,113].

The presence of endocrine cells in tumor metastases confirms the malignant nature of these cells. The authors also documented a significant correlation between the histological type of the tumor and the biological properties of the molecules it produces, *i.e.*, MLT, serotonin, and somatostatin, all having an antiproliferative activity [114]. These substances were more frequent in the more differentiated tumors such as adenocarcinomas and squamous cell carcinomas with keratinization, while catecholamine, histamine, insulin, gastrin, and TSH, substances inducing proliferative activity, were usually more frequent in tumors that have a higher proliferative index, those that are more aggressive and less differentiating, such as solid tumors and squamous cell carcinomas without keratinization. These data suggest that the *in situ* production of MLT and of the relative APUD peptides in non-endocrine tumors plays a determining role in the autocrine mechanisms of tumoral homeostasis, promoting, slowing down, inhibiting or preventing progression and metastasization.

Additional confirmation comes from studies relative to the significant increase of cells that are immunopositive for MLT in non-metastatic human breast cancer [115]. Confirmation is also provided by studies on the oncostatic effect of MLT on the mammary gland in transgenic mice with *N*-ras proto-oncogene, which have demonstrated that MLT reduces the incidence of hyperplastic alveolar nodules and the presence of *N*-ras protein in focal hyperplastic lesions [47].

Maestroni and Conti found concentrations of MLT in breast cancer cells triple those of the serum rate of healthy subjects [116].

Epithelial and APUD cells originate from common stem cells and the presence of APUD cells in non-endocrine tumors depends on the level of malignant transformation. Hormonal secretion in tumors originating from non-endocrine cell aggregates is not an autonomous sign, but a genetically induced element, caused by cell genesis and differentiation. This process is directly associated with cell growth, division and differentiation potential, and the prognostic aspect deriving from the identification of the chemical composition and biological hormonal activity produced by these tumor cells should therefore not be underestimated.

### 4.4. Platelets and the APUD System

Platelets can be considered omnipresent, multifactorial and itinerant elements of a plastic and ubiquitary APUD system, with its content of serotonin (5-TH) and norepinephrine, acetylcholine and epinephrine, MLT, NAT and HIOMT, metabolic byproducts and deposit of adenosine (AMP, ADP, ATP). Platelets sometimes act like a melatonergic and dopaminergic, serotonergic and adrenergic neuron, depending on local conditions and the working nature of the nuclei. Platelets can absorb and store 5-TH; they can also synthesize MLT since they also contain 5-TH-decarboxylase [117,118].

There is a large quantity of pharmacological data indicating considerable functional affinity and complementary action between the platelets and neurons of the serotonergic system. This function of the platelets, which release their deposits of 5-TH and expel material from their granules when activated by appropriate stimuli has been considered very similar to the release of neurotransmitters by central neurons. The platelet release reaction and the secretion activity together act as a model for the release of central serotonergic and adrenergic neurons [119–121].

### 4.5. Melatonin’s Action on Microtubules

MLT carried out its anticancer activity also on the intercellular gap junctions that mediate communication between adjacent cells and are closely connected to the mechanisms that condition cellular growth. A study by Kojma *et al.* on rat hepatocytes demonstrated the induction by MLT of the CX32 gap junction protein [122–124].

The process of tubulin polymerization may also be one of the intercellular objectives of the action of MLT on tumor cells. Meléndez *et al.* demonstrated that physiological concentrations of MLT induce an increase of microtubules in NIE-115 neuroblastoma cells, and that this effect is due to an increase of the polymerization status of tubulin [80,125,126].

## 5. Melatonin and Tumor Treatment

### 5.1. Clinical Significance and Therapeutic Application

Several clinical trials have examined the therapeutic usefulness of melatonin in different types of cancer. The conclusion is that the use of melatonin as an adjuvant therapy seems to be very useful for early stages than for advanced and metastatic cancers [127–130]. Use a strongly helpful aid for side effects caused by chemotherapy and radiotherapy administration was also reported [61,131–133]. Moreover, all the investigations mentioned documented the very low toxicity of melatonin over a wide range of doses. On the basis of this preliminary studies, it seems that melatonin administration may be beneficial for oncological subjects [134–137].

### 5.2. Future Prospects after 30 Years of Research

The absolute priority of the anticancer use of melatonin belongs to Luigi Di Bella, who believed that the antiblastic activity of MLT was not limited to the aforementioned mechanisms of action, nor to the biochemistry of MLT or of other pineal methoxyindoles [138,139]. It has also been shown that MLT can reach the nucleus of the megakaryocyte and carry out a similar action to cytochalasin B, both in inhibiting the process of endoduplication and in increasing nuclear polyploidy [140,141].

Di Bella was the first to identify the fundamental and primary role of MLT in providing the phosphor esters of AMP, ADP and ATP [69].

This concept is fundamental for the relationship and close connection with the school of thought led by Goldberger, Epstein and Anfinsen, which also allows the possibility of self-assembly and that the protein can spontaneously restore its three-dimensional structure with full biological activity (protein folding) [142].

It could be the same or another protein that influences the intermolecular reactions. Some proteins act as molecular chaperones and by hydrolyzing ATP they activate the folding of protein structures that are otherwise inert [143,144]. The mechanism of action was explained by Ellis, who identified the chaperonins as sequestrating agents containing the folded individual protein structures in the Anfinsen cage [145,146]. According to Luigi D Bella, in neoplastic biology the action of the chaperonins should prevalently take place through the hydrolysis of ATP, ADP, and AMP bonded with adenosine or MLT [69,98,99] (Figure 1).

### 5.3. Indications Regarding the Proposed Dosage of Melatonin in Prevention

The dosage of MLT in prevention vary according to age, sex, familiarity, current and/or previous diseases, type of activity carried out with exposure to cancerogenous molecules and/or relative to the duration of exposure and the intensity of magnetic fields. The dosage also considers nocturnal exposure at work to artificial light with relative inhibition of the pineal secretion of MLT [120,147,148]. To reinforce the immune system through the increase of interleukin 2, MLT, together with retinoids, vitamin E, vitamin C, and vitamin D3 improves the antifective and antiblastic immune responses; the dosage in prevention must therefore be increased in immunodepressed subjects [79].

In children, dosage starts with the evening administration of 2 mg, increasing gradually after adolescence. In adult males of average weight and age, 4–5 mg can be administered in the evening; slightly less in fertile age females: 2–3 mg. After the age of 50 years, especially in post-menopausal women due to MLT’s ability to inhibit potentially cancerogenous molecules, in addition to GH, such as prolactin, estrogens and androgens, the dose can be gradually increased to 10 mg [64,149–151].

In the presence of fibrocystic breasts, ovarian cysts, myomas, uterine fibromas, or endometrial thickening, 15–20 mg can be administered depending on the intensity of the disease.

Similar doses can be administered also in the case of prostatic hypertrophy. In these male and female often precancerous situations, the synergism with prolactin inhibitors and retinoids solubilized in vitamins E and D3 has proved particularly useful for the documented high receptorial expression in the prostate, uterine microfibromas and breast, in addition to MLT and D2, VDR and RXR receptors. The same doses and synergism apply to nodules of the thyroid (normal or hyperfunctioning) which also have a similar receptorial expression. In the thyroid, the decrease in the volume of the nodules is accelerated by the use of somatostatin and also low doses (0.1–0.2 mg) of octreotide.

### 5.4. Indications Regarding the Proposed Dosage of Melatonin in the Treatment of Tumors

Although the ideal dose of melatonin has not yet been standardized, some clinical studies, in addition to our own results, have shown that daily oral doses of 20–40 mg (distributed evenly throughout the day with greater concentrations in the evening) [149–151], up to a maximum of 1000 mg of Melatonin administered slowly and intravenously during the day, are perfectly well tolerated, with useful and beneficial effects for the patients [152,153]. In over 42 years of experience in the clinical use of MLT by Luigi Di Bella, Giuseppe Di Bella and others, the dose has been gradually increased, without toxicity or significant side effects, except for temporary drowsiness reported by some patients, generally at the start of the treatment, and very rarely making it necessary to reduce the dose. Patients diagnosed at an initial/early stage of the disease can be given 30 mg of melatonin orally, and the maximum dose is also advised for patients with sleeping disorders. Since numerous clinical studies have shown that patients with an advanced/terminal stage of the disease or who no longer respond to traditional treatments can benefit from the administration of high doses of MLT; these patients could consider taking a supplement of MLT of 100 mg.

The hydrogen bond with adenosine (Figure 1) improves its bioavailability, makes it hydrosoluble and forms the base molecule for the synthesis and diffusion of phosphor esters of AMP, ADP and ATP, which have a significant role in physiological and neoplastic biology, as previously described.

## 6. Conclusions

In the present marked cultural decline, muffled by the superficial culture of everyday events, rational technological simplification is not always able to compensate for the burden of progressive ignorance. In the health sector, costs have soared because too many unnecessary expensive, if not harmful, tests are performed, because patients are flooded with technically and aesthetically perfect but useless, if not toxic drugs, and because expensive and pointless hospital stays are prolonged.

Ignorance of the real disease and appropriate remedies to cure it is the primary cause of incorrect care, patient malaise and disproportionate balance sheets.

The cancer problem can be satisfactorily and largely resolved only by optimization, courageously eliminating the irrationality of the past, reaching out towards a future in which cancer becomes a normal occurrence of future human existence, because means will have been found and correctly applied.

If physicians remember the fundamental saying “*Primum non nocere*” addressed to the patient, but also and above all to their colleagues, then the health sector would undoubtedly improve. No profession is perhaps founded so much on morals as the medical profession. There are numerous famous sayings that commend this profession, some evasive and deceiving. Loving one’s fellow man would be enough, aspiring to change the expression of pain into a progressive image of acceptable prognosis, to achieve the aim. It is not too much to expect the highest level of general morality from one’s doctor.

The desire to hand on, at least in part, the ideas of Luigi Di Bella was the stimulus for publishing this review, in the hope that one day his dream would become reality. The basis for his ideas is to consider cancer as a form of life, a life that he defined as “potent, overwhelming, parasitic and anarchic”. It is necessary to combine a series of substances that can act in a centripetal way on the tumor cells and that can have an effect, from time to time, simultaneously or successively, on the myriad of biological reactions that are responsible for the life of these cells. This gave rise not to a substance but to a method [42,153].

Considering that: (a) the treatment of solid tumors is based essentially on surgery; (b) there are no statistics in the literature regarding solid tumors cured in a stable way by chemotherapy alone and that if a tumor exceeds the surgical limits, chemotherapy and/or monoclonal antibodies are unable to cure it; (c) the results of the current medical treatments for cancer are still extremely limited and are often temporary [154,155]; (d) chemotherapy treatments are penalized by toxicity, sometimes fatal [156,157]; (e) due to its mutagenic effect, chemotherapy is able to select strains of increasingly more resistant and aggressive tumor cells [158]; and (f) the increase in resistance and aggression of tumor cells can also be induced by radiotherapy [159]; we wanted to draw attention to the use of MLT in oncology, believing that, by combining the documented anticancer properties with an antitoxic, trophic, immunostimulating, differentiating, radioprotective and radiosensitizing effect, the oncotherapuetic possibilities of this pineal indole are still greatly underestimated [160–166].
